# Comparison of seven diagnostic tests to detect Trypanosoma cruzi infection in patients in chronic phase of Chagas disease

**Published:** 2014-06-30

**Authors:** Luisa Fernanda Duarte, Oscar Flórez, Giovanna Rincón, Clara Isabel González

**Affiliations:** Molecular Immunology and Epidemiology Group, GIEM, Facultad de Salud, Universidad Industrial de Santander, Bucaramanga, Colombia

**Keywords:** Chagas disease, Trypanosoma cruzi, Enzyme-linked immunosorbent assay, diagnosis, recombinant proteins, polymerase chain reaction, Colombia

## Abstract

**Objective:**

To compare the diagnostic performance of seven methods to determine *Trypanosoma cruzi* infection in patients with chronic Chagas disease.

**Methods:**

Analytical study, using the case-control design, which included 205 people (patients with Chagasic cardiomyopathy, n= 100; control group, n= 105). Three enzyme linked immunosorbent assays, one indirect hemagglutination assay and one immunochromatographic test were assessed. Additionally, DNA amplification was performed via the PCR method using kinetoplast and nuclear DNA as target sequences. For the comparative analysis of diagnostic tests, the parameters used were sensitivity, specificity, positive and negative predictive values, Receiver Operator Characteristic (ROC), positive and negative likelihood ratio, as well as κ quality analysis.

****Results**:**

The commercial Bioelisa Chagas test showed the highest sensitivity (98%), specificity (100%), and positive and negative predictive values; ​​additionally, it had the highest discriminatory power. Otherwise, the amplification of *T. cruzi* DNA in blood samples showed low values of sensitivity (kinetoplast DNA = 51%, nuclear DNA = 22%), but high values of specificity (100%), and moderate to low discriminatory ability.

**Conclusion:**

The comparative analysis among the different methods suggests that the diagnostic strategy of *T. cruzi *infection in patients with chronic Chagas disease can be performed using ELISA assays based on recombinant proteins and/or synthetic peptides, which show higher diagnosis performance and can confirm and exclude the diagnosis of *T. cruzi* infection. The molecular methods show poor performance when used in the diagnosis of patients with chronic Chagas disease.

## Abstract (es)

**Objetivo:**

Comparar la capacidad diagnóstica de siete métodos para determinar infección por *Trypanosoma cruzi,* en pacientes con enfermedad de Chagas crónica.

Estudio analítico de casos y controles, que incluyó 205 personas (pacientes con miocardiopatía chagásica, n= 100; grupo control, n= 105). Se evaluaron tres inmunoensayos enzimáticos, una hemaglutinación indirecta y una inmunocromatografia. Adicionalmente, se realizó amplificación de ADN de *T. cruzi *por reacción en cadena de la polimerasa utilizando como secuencias diana ADN de kinetoplasto y nuclear. Para el análisis comparativo de las pruebas diagnósticas, los parámetros utilizados fueron sensibilidad, especificidad, valores predictivo positivo y negativo, análisis ROC, razón de verosimilitud positiva y negativa, así como análisis de calidad κ.

La prueba de Chagas Bioelisa mostró la mayor sensibilidad (98%), especificidad (100%) y valores predictivos positivo y negativo; además tuvo el mayor poder discriminatorio. En contraste, los ensayos de amplificación de ADN de *T. cruzi* mostraron baja sensibilidad (ADN de kinetoplasto = 51%, ADN nuclear = 22%), alta especificidad (100%) y de moderada a baja capacidad discriminatoria.

**Conclusión:**

El análisis comparativo entre los métodos sugiere utilizar como estrategia diagnóstica en pacientes crónicos con enfermedad de Chagas, los ensayos de ELISA con proteínas recombinantes y/o péptidos sintéticos por mostrar un rendimiento diagnóstico superior y tener la capacidad de confirmar y descartar el diagnóstico de infección por *T. cruzi*. Los métodos moleculares muestran pobre rendimiento para ser utilizados en el diagnóstico de pacientes en fase crónica con enfermedad de Chagas.

## Introduction

Chagas disease (CD) is caused by infection with the intracellular protozoan parasite *Trypanosoma cruzi*. The World Health Organization estimated that approximately 8-millions people in Latin America are affected 1. However, due to increasing migration of Latin Americans around the world, this pathology should now be considered a global disease 2. In Colombia, *T. cruzi *infection prevalence is around 5%, corresponding to 700,000 people 3, and in some areas of the department of Santander the seroprevalence is about 50% 4. The clinical manifestations of CD include an acute and a chronic phase, which presents a wide spectrum of clinical manifestations including cardiac, digestive and neurological forms. Nevertheless, only approximately 20-30% of infected individuals develop chronic Chagasic cardiomyopathy and/or megaesophagus/megacolon 2.

The diagnosis of infection with *T. cruzi* is complex, especially during the chronic phase, due to the lack of symptoms and the low or intermittent parasitemia 2 that leads to direct parasitological methods having low sensitivity. For this reason, the diagnosis is based on serological methods which detect the presence of specific antibodies directed against antigens of *T. cruzi *combined with clinical and epidemiological findings. However, serological tests present high sensitivity, but lack specificity because of antigenic cross-reactivity with other parasites like *Leishmania sp*. and *T. rangeli* 5. In this scenario, the Pan American Health Organization (PAHO) suggested that at least two assays based on different techniques may be used in parallel to increase diagnostic accuracy because a single assay is not considered sufficiently sensitive and specific 6. But, this strategy has led to increased inconclusive results that hinder clinical management of these cases. Additionally, correct diagnosis is not only a priority to identify individuals, who should receive appropriate treatment, but also to reduce and prevent the risk of transmission through blood transfusion and/or organ transplant. 

Immunological methods are based on enzyme-linked immunosorbent assay (ELISA), indirect hemagglutination assay (IHA), immunofluorescence indirect assay (IFI), immunoblotting assay (IB), and immunochromatographic assay (IC). Most assays use crude lysates of the parasite as antigen; however the use of recombinant proteins and / or synthetic peptides have been described that increase the specificity of the tests 7 ^-^ 9. Even though immunological methods are used in the diagnosis of *T. cruzi *infection, molecular methods provide an alternative especially in cases of doubtful serology 10 ^,^ 11. These methods are based mainly on amplification by polymerase chain reaction (PCR). Nevertheless, nested-PCR assay (N-PCR) 10, quantitative real-time PCR assay (qRT-PCR) 11, and oligochromatography assay (OligoC) 12 have been performed to improve detection of *T. cruzi* DNA. Given the heterogeneity of the performance reported of tests available for diagnosis, the aim of this study was to compare the overall accuracy of the serological and molecular methods to detect *T. cruzi* infection in patients with chronic Chagas disease.

## Materials and Methods

###  Study subjects and samples

The study is an analytical study, using the case-control design, which included a total of 205 people. In the study, individuals were chosen from a database of approximately 2,000 patients who had been recruited for a molecular epidemiology study on Chagas disease, conducted by our research group for the past 10 years. The database has epidemiologic, clinical, and laboratory information from each participant. The epidemiologic data collection was carried out face-to-face by trained interviewers independently from medical staff who filled out a questionnaire. The clinical diagnosis was established by an independent consensus panel, consisting of two clinicians, who are experts in the field of cardiology. In order to know the diagnostic value of each serological and molecular method for *T. cruzi *detection, and because a gold standard test for diagnosis of CD does not exist, the selection of individuals was performed by combining epidemiological and clinical characteristics. Thus, and as inclusion criteria, people from rural areas where the endemicity level is high, and who have cardiomyopathy clearly compatible with CD by electrocardiogram, echocardiogram and 24-h Holter comprised the group of patients with Chagasic cardiomyopathy (n= 100), whereas people without cardiac signs and symptoms, and who come from a non-endemic urban area made up the control group (n= 105). Furthermore, all individuals lived in these areas for 10 and more years. Sample collection was as follow for each subject, three blood samples were collected; one of these (6 mL) was used to acquire serum; and the other two (4 mL each and EDTA-anticoagulant) were used to isolate genomic DNA from buffy coat. The time gap and storage temperature between blood collection and DNA extraction was 48 h at 4° C. Serum and DNA samples were stored until tested by freezing at -70° C and -20° C, respectively. These samples were used to evaluate the diagnostic performance of serological and molecular methods for detection of *T. cruzi* infection. Laboratory testing was carried out by two professional microbiology experts, who were masked for all information related to the individuals. Two researchers, who were also masked for all information related to the individuals, reviewed the results of laboratory testing. The individual panel members reviewed each laboratory test before meeting to agree on a final testing result. All laboratory tests were correctly allocated, with 100% concordance among the members of the panel.

### Serological methods

Serum anti-*Trypanosoma cruzi* antibodies were determined by in-house and recombinant ELISA, IHA and IC tests. 

**Figure 1.**
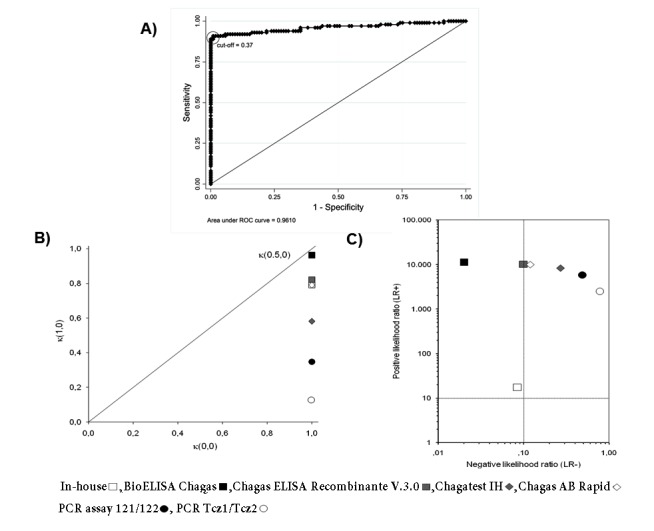
Analysis of diagnostic tests to detect *Trypanosoma cruzi *infection in patients with chronic Chagas disease. **A) **ROC curve of in-house ELISA. It shows the analysis of sensitivity and specificity at different cut-off values of in-house ELISA compared with the diagnosis of Chagas disease by clinical (electrocardiogram, echocardiogram and 24-h Holter), epidemiological evaluation and laboratory findings. **B) **Quality of sensitivity, specificity, and efficiency of each serological and molecular diagnostic tests. The optimum would be a test with values at the top right and on diagonal. κ(1,0) = quality of sensitivity, κ(0,0) = quality of specificity, κ(0.5,0) = quality of efficiency. **C) **Positive Likelihood ratio (LR+) and negative Likelihood ratio (LR-) of each serological and molecular diagnostic test. Test in right upper quadrant only confirms the diagnosis and in left lower quadrant only excludes the diagnosis. The optimum would be a test in the left upper quadrant because it confirms and excludes the diagnosis.

The in-house ELISA was carried out in 96-well microtiter plates (Dynatech micro ELISA system; Germany) with soluble extract of an autochthonous strain of *T. cruzi *I epimastigotes (MHOM/CO/06/338).The plates were coated with 100 µL per well of 2.0 µg mL^-1^ of antigen diluted in buffer carbonate-bicarbonate, pH 9.6, and incubated overnight at 4° C. After that, the plates were washed with Tween 20 (0.05%) in phosphate buffered saline (137 mM of NaCl, 2.68 mM of KCl, 1.47 mM of Na_2_HPO_4_ and 9.03 mM of KH_2_PO_4_·2H_2_O), pH 7.4 (PBS-T20). The remaining binding sites were blocked with 2% skim milk in PBS-T20. Each sample was tested in duplicated wells using 100 µL of serum diluted at 1:800 in PBS-T20. The plates were incubated for 1 h at room temperature and washed again. Promptly, 100 µL of anti-human polyvalent immunoglobulins (α, γ and μ-chain specific) alkaline phosphatase conjugated: Cat no. A3313 (Sigma-Aldrich, Inc.; USA), diluted at 1:6,000 in PBS-T20, were added and incubated for 1 h at 37° C and then washed again. After incubation and washing, 100 µL of 1 mg mL^-1^ p-nitrophenyl phosphate (Sigma-Aldrich, Inc.; USA) in 10% diethanolamine buffer, pH 9.7, were added and the plates were incubated for 25 min at room temperature. Finally, the reaction was stopped with 50 µL of 3 M NaOH. The optical density (OD) at 410 nm was measured on a microplate reader Model MR550 (Bio-Rad Laboratories, Inc.; USA). A sample was considered positive if the OD was equal to or greater than 0.37, this cut-off was estimated based on ROC curve analysis. The optimum cut-off was defined as the value that maximized the area under the ROC curve (Fig. 1A).

All samples were also tested by Bioelisa Chagas (Biokit; Spain), which uses synthetic peptides TcD, TcE, PEP2 and TCLi1-2 as antigen and by Chagatest ELISA Recombinante V.3.0 (Winer Lab.; Argentina), which uses recombinant proteins Ag1, Ag2, Ag13, Ag30, Ag36, and SAPA as antigen. Other tests used were Chagatest IHA (Winer Lab.; Argentina), which uses sheep erythrocytes sensitized with parasite lysate as antigen; and IC test which includes the recombinant antigens H49 and 1F8 as antigen (Chagas AB Rapid, Standard Diagnostics; Korea). All determinations of commercial kits were performed according to manufacturer's instructions.

### Molecular methods 

Genomic DNA was isolated from buffy coat of 4 mL of EDTA-anticoagulated blood samples using the standard salting-out technique 13. *T. cruzi *nuclear and kinetoplast DNA were amplified by PCR method using the PTC-200 DNA Engine^®^ Thermal Cycler (Bio-Rad Laboratories, Inc.; USA). Detection limit of the *T. cruzi *DNA for optimized PCR protocols was estimated on 10 parasites per 100 µg μL^-1 ^of total DNA isolated. This concentration was determined by mixing EDTA-anticoagulated blood samples from a healthy person (non-infected with *T. cruzi*) with 1 mL of *T. cruzi* I epimastigotes. The mixes tested were: 1,000, 100, 10, 1, 0.1, 0.01, and 0.001 parasites in 4 mL of whole blood. The genomic DNA was isolated from buffy coat as mentioned above and different DNA concentrations were tested in each PCR assay. All experiments were performed in triplicate on three independent occasions.

The repeat tandem sequence of nuclear DNA (nDNA) of *T. cruzi* was amplified by primers Tcz1 (5'-CGA GCT CTT GCC CAC ACG GGT GCT-3') and Tcz2 (5'-CCT CCA AGC AGC GGA TAG TTC AGG-3'), which amplify a (188-pb fragment by 30 cycles (94°C for 30 s, 55° C for 30 s, 72° C for 30 s). Each PCR contained 0.5 µM of each primer, 2 mM of MgCl_2_, 200 µM of dNTPs, 1X Taq buffer, and 1 U of *Taq* DNA polymerase (Invitrogen Brazil Ltda.; Brazil). The variable region of the minicircle kinetoplast DNA (kDNA) of *T. cruzi* was amplified by primers 121 (5'-AAA TAA TGT ACG GGK GAG ATG CAT GA-3') and 122 (5'-GGT TCG ATT GGG GTT GGT GTA ATA TA-3'), which amplify a (330-pb fragment by 35 cycles (94° C for 1 min, 63.5° C for 1 min, 72° C for 1 min). Each reaction contained 0.5 µM each primer, 4.5 mM of MgCl_2_, 200 µM of dNTPs, 1X Taq buffer and 1.25 U of *Taq* DNA polymerase (Invitrogen Brasil Ltda.; Brazil). The PCR conditions of amplification were carried out with 800 ng of template DNA in a total volume of 20 μL. The PCR products were analyzed by electrophoresis on 2% agarose gel stained with ethidium bromide in 1X TAE buffer, each amplicon was recognized according to its size by using the molecular weight size marker XIV (Roche Applied Science; USA). All DNA extraction steps and reaction mixtures used for PCR assays were monitored and compared with positive and negative controls; the positive controls included DNA isolated from *T. cruzi *Silvio X-10 strain and DNA isolated from blood infected with two *T. cruzi *I strains (MHOM/CO/07/REM and MHOM/CO/07/338); whereas the negative controls included DNA isolated from *T. rangeli*, *Leishmania panamensis*,* Toxoplasma gondii*, *Crithidia lucilliae *and DNA isolated from blood non-infected with *T. cruzi*.

### Ethical consideration

This study complies with current Colombia laws and fulfilled all criteria required by the Medical Code of Ethics and declaration of Helsinki. The Ethics Committee at Universidad Industrial de Santander approved this study and a written informed consent was obtained from all participants.

### Data analysis

 For all commercial kits, each value of sensitivity, specificity, positive predictive value (PPV), negative predictive value (NPV), and ROC, as well as its own confidence intervals were estimated by using cut-off values recommended by manufacturers. Similarly, LR+ and LR- were estimated, as well as quality of sensitivity (κ (1,0)), specificity (κ (0,0)), and efficiency (κ (0.5,0)) 14 ^,^ 15. Statistical analyses were performed using the software STATA version 10 (STATA Corp., College Station, Texas; USA).

## Results

This study was performed between June 2010 and July 2011. Epidemiologic survey, clinical diagnosis, and sample collection have been done since 2001. Standardization of laboratory tests used in the study was performed from August 2010 to November 2010, and laboratory testing was carried out from December 2010 to March 2011. The clinical and demographic characteristics of the study population is: the cases group was comprised of 100 patients with Chagasic cardiomyopathy, 47 females and 53 males, with mean age of 50.6 ± 6.4 years; and control group was made up of 105 healthy subjects, 63 females and 42 males, with mean age of 23.7 ± 3.3 years. All participants selected fulfilled the criteria for inclusion in the study. 

Conditional and unconditional probabilities for each serological and molecular test, as well as their analysis of quality are listed in Table 1. Among all the tests performed in this study, Bioelisa Chagas showed the highest values of sensitivity, specificity, PPV, NPV, and discriminatory ability (Table 1 and Fig. 1B). Otherwise, the PCR assays used to detection *T. cruzi* DNA in blood samples showed low sensitivity values, but high specificity values; however, they showed moderate to low discriminatory ability (Table 1 and Fig. 1B). In addition, correlation analysis of serological methods was higher than molecular methods, which were moderate to low (Table 2).

**Table 1.**
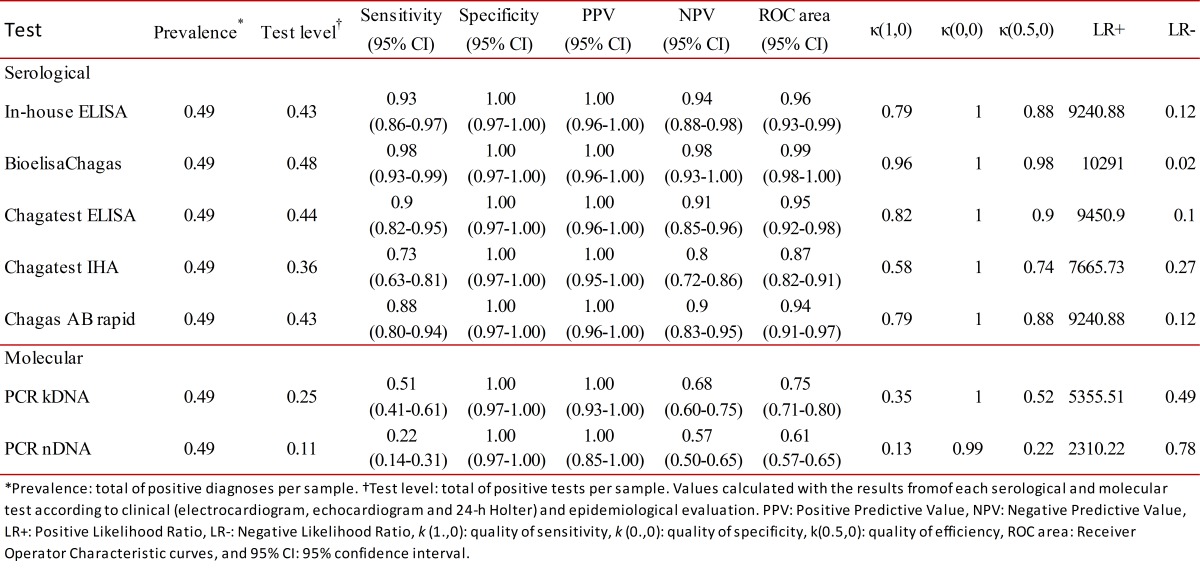
Diagnostic results and performance of serological and molecular tests

**Table 2:**
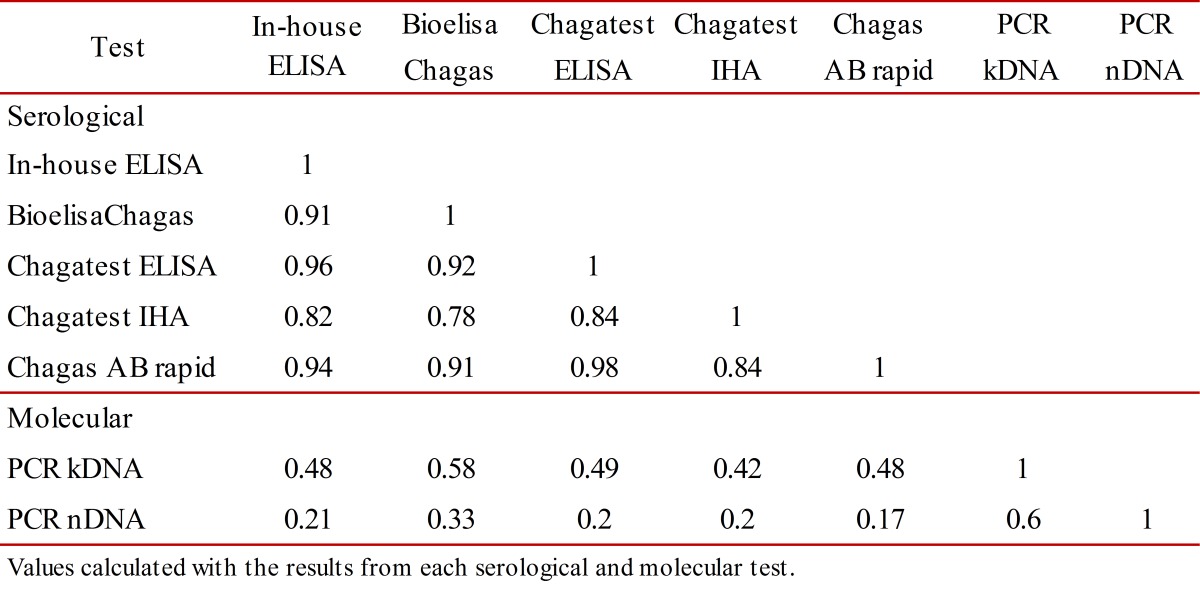
Diagnostic correlation of serological and molecular tests

Also, analysis of LR+ and LR- showed that Bioelisa Chagas and Chagatest ELISA Recombinante V.3.0 can confirm and exclude the diagnosis of CD; whereas, Chagatest IHA, Chagas AB Rapid and in-house ELISA, as well as the PCR assays performed with the primer sets Tcz1/Tcz2 and 121/122, can only confirm the diagnosis of CD (Fig. 1C).

## Discussion

In the chronic phase of the Chagas disease, diagnosis is based on the presence of anti *T. cruzi *antibodies due to the absence or low parasitemia; therefore, serologic tests like ELISA, IFA, and IHA are commonly used. In order to solve the drawbacks of false positive and false negative results with conventional serological test, developed non-conventional serological assays have been developed using recombinant proteins of *T. cruzi*, which present values of sensitivity and specificity close to 100% 7 ^-^ 9 ^,^ 16. Despite these advances, and since currently no reference test is available, the PAHO recommends using two tests based on different principles to detect different antigens 6. However, this guideline has increased discordant results and difficulties in diagnosis. In addition, there are numerous commercially available tests, but significant heterogeneity related to the accuracy of these tests makes it difficult to select the most appropriate to ensure diagnosis in endemic areas.

Experimental evidence from this study showed that Bioelisa Chagas and Chagatest ELISA Recombinante V 3.0 showed the highest sensitivity and specificity values, as well as PPV and NPV. Moreover, they present good discriminatory ability and high quality of sensitivity and specificity; in addition to the capacity confirm and exclude the diagnosis of *T. cruzi* infection in patients in the chronic phase of Chagas disease (Table 1, and Figs. 1B and C). However, even though the correlation level is high (Table 2), the results obtained by using Bioelisa Chagas were better than with Chagatest ELISA Recombinante V 3.0, as reported in previous studies in Colombia, in which the latter showed 95% sensitivity 17. This can be explained by differences in composition and mixtures of synthetic peptides or *T. cruzi* recombinant proteins*. *Thus, Bioelisa Chagas includes synthetic peptides TcD, TcE, PEP2 and TCLi1-2 (www.biokit.com), which model immunodominant antigenic epitopes of *T. cruzi* 18; whereas, Chagatest ELISA Recombinante V 3.0 includes recombinant proteins Ag1, Ag2, Ag13, Ag30, Ag36 and SAPA (www.wiener-lab.com.ar). The sensitivity and specificity characteristic of each peptide/protein and their mixtures were previously reviewed by Jose Franco da Silveira 9. Nevertheless, it is important to note that both tests show high correlation levels between them; moreover, they exhibit antigens mainly recognized by IgM antibodies, such as TCLi1-2 and SAPA. However, the antigen-antibody reaction in Bioelisa Chagas is identified by anti-human IgG and IgM, whereas in Chagatest ELISA Recombinante V 3.0 it is only identified by anti-human IgG. Otherwise, the immunochromatographic Chagas AB Rapid assay is a rapid diagnostic test that uses the recombinant antigens H49 and 1F8, which have shown sensitivity and specificity values from 97 to 100% 16 ^,^ 19. This evidence may explain the good results obtained in sensitivity, specificity, PPV, NPV, quality of sensitivity, quality of specificity, and discriminatory ability (Table 1). In addition, its simplicity and ease interpretation make it very useful in rapid diagnosis of infection with *T. cruzi* in field studies. However, for subjects with negative results it will be necessary to use any of the other tests to reject the diagnosis of *T. cruzi* infection (Table 2). On the other hand, the in-house ELISA and IHA exhibit good values of sensitivity, specificity, PPV, and NPV. However, in-house ELISA has better values of sensitivity and NPV than Chagatest IHA, while, Chagatest IHA has better values of specificity and PPV than in-house ELISA (Table 1); furthermore, in-house ELISA presented higher discriminatory ability and capability of confirm and exclude the diagnosis of *T. cruzi* infection (Table 1, and Figs. 1B and C). Nevertheless, these tests exhibited false positive and false negative results, which could be solved or improved by using antigenic preparations of trypomastigotes and/or amastigotes of autochthonous strains of *T. cruzi*.

Detection of *T. cruzi *in human blood samples by DNA amplification using methods based on PCR has been applied to diagnose CD in patients who have progressed to the chronic phase. But, given that during this phase the number of parasites circulating in the peripheral blood is low or intermittent, the PCR-based methods have sensitivities in the order of 45-65%, while specificity remains close to 100% 5 ^,^ 20 ^,^ 21. Nonetheless, although the target sequences used in this study have a high number of copies in the *T. cruzi* genome (5,000 to 10,000 copies of kDNA and (10% of nDNA per parasite) 22 ^,^ 23, the results showed moderate to low values of sensitivity and quality of sensitivity for PCR essays carried out with primers 121/122 and Tcz1/Tcz2 (Table 1 and Fig. 1B). These results could be explained, at least partly by the availability of DNA template in the reaction mixture, which could be related to the type of *T. cruzi *strain. Thus, differences are noted in the number of copies of satellite DNA targets among *T. cruzi *strains, which are more abundant in *T. *cruzi II than *T. cruzi *I 24, as well as, differences in the level of parasitemia, which is higher in infection by *T. cruzi *I compared to *T. cruzi* II 20. These findings are interesting because in Colombia *T. cruzi *I is the predominant group in both domestic and sylvatic cycles, nonetheless, evidence has been presented of *T. cruzi *II infection in patients with Chagasic cardiomyopathy 25. These results show that patients who have a positive PCR result can be diagnosed as *T. cruzi *infected, but patients with negative PCR result, it will be necessary to use any of the other tests to reject the diagnosis of *T. cruzi* infection. This, indicate that molecular tests can confirm the diagnosis but not exclude it (Fig. 1C). In fact, these molecular tests showed moderate to low correlation with the other tests (Table 2).

In conclusion, our experimental evidence suggests that the strategy of diagnosing *T. cruzi *infection in patients who have progressed to the chronic phase of CD can be done by using Bioelisa Chagas or Chagatest ELISA Recombinante V 3.0, which not only showed better diagnostic performance, but can also confirm and exclude the diagnosis of *T. cruzi* infection. Moreover, Chagas AB Rapid could be used in cases where rapid diagnosis is necessary. Finally, the molecular assays can be used to confirm the diagnosis; however, due to the low quality of sensitivity, specificity and discriminatory ability, it is important to use any of the other tests to reject the diagnosis of *T. cruzi* infection.
